# Glutathione Peroxidase 1 Protects Against Peroxynitrite-Induced Spiral Ganglion Neuron Damage Through Attenuating NF-κB Pathway Activation

**DOI:** 10.3389/fncel.2022.841731

**Published:** 2022-03-23

**Authors:** Xue Wang, Yuechen Han, Fang Chen, Man Wang, Yun Xiao, Haibo Wang, Lei Xu, Wenwen Liu

**Affiliations:** ^1^Department of Otolaryngology-Head and Neck Surgery, Shandong Provincial ENT Hospital, Cheeloo College of Medicine, Shandong University, Jinan, China; ^2^Shandong Institute of Otorhinolaryngology, Jinan, China

**Keywords:** glutathione peroxidase 1, spiral ganglion neuron, peroxynitrite, oxidative stress, nuclear factor-kappa B, hearing loss

## Abstract

Glutathione peroxidase 1 (GPX1) is a crucial antioxidant enzyme that prevented the harmful accumulation of intra-cellular hydrogen peroxide. GPX1 might contribute in limiting cochlear damages associated with aging or acoustic overexposure, but the function of GPX1 in the inner ear remains unclear. The present study was designed to investigate the effect of GPX1 on cochlear spiral ganglion neurons (SGNs) against oxidative stress induced by peroxynitrite, a versatile oxidant generated by the reaction of superoxide anion and nitric oxide. Here, we first found that the expression of GPX1 in cultured SGNs was downregulated after peroxynitrite exposure. Then, the GPX1 mimic ebselen and the *gpx1* knockout (*gpx1*^–/–^) mice were used to investigate the role of GPX1 in SGNs treated with peroxynitrite. The pretreatment with ebselen significantly increased the survived SGN numbers, inhibited the apoptosis, and enhanced the expression of 4-HNE in the cultured SGNs of peroxynitrite + ebselen group compared with the peroxynitrite-only group. On the contrary, remarkably less survived SGNs, more apoptotic SGNs, and the higher expression level of 4-HNE were detected in the peroxynitrite + *gpx1*^–/–^ group compared with the peroxynitrite-only group. Furthermore, rescue experiments with antioxidant N-acetylcysteine (NAC) showed that the expression of 4-HNE and the apoptosis in SGNs were significantly decreased, while the number of surviving SGNs was increased in peroxynitrite + NAC group compared the peroxynitrite-only group and in peroxynitrite + *gpx1*^–/–^ + NAC group vs. peroxynitrite + *gpx1*^–/–^ group. Finally, mechanistic studies showed that the activation of nuclear factor-kappa B (NF-κB) was involved in the SGNs damage caused by peroxynitrite and that GPX1 protected SGNs against peroxynitrite-induced damage, at least in part, *via* blocking the NF-κB pathway activation. Collectively, our findings suggest that GPX1 might serve as a new target for the prevention of nitrogen radical-induced SGNs damage and hearing loss.

## Introduction

In mammals, cochlea spiral ganglion neurons (SGNs) are the primary sensory neurons on the auditory conduction pathway. SGNs transmit complex acoustic information from sensory hair cells (HCs) to the second-order sensory neurons in the cochlear nucleus for sound processing. The survival of SGNs is essential for hearing preservation and, conversely, as SGN cannot regenerate spontaneously, the impairment and irreversible loss of SGNs result in permanent sensorineural hearing loss (SNHL) (Liu et al., 2019a; Leake et al., 2020; Pavlinkova, 2020), which seriously affects the human social and cognitive development. Multiple stimuli, such as excessive noise, ototoxic drugs, hereditary defects, and aging process, can cause SGNs damage. Although the pathogenic mechanisms by which these factors lead to SGN damage are various, researches have documented that the oxidative stress is a basic mechanism involved in SGNs death (Liu et al., 2011, 2012, 2019b,^2021^; Xiong et al., 2011; Yamasoba et al., 2013; Wang et al., 2019, 2021). Oxidative stress is the disturbance in the balance between the generation of free radicals, such as reactive oxygen species (ROS) and reactive nitrogen species (RNS), and antioxidant defenses, and can cause oxidative damage to diverse cellular components, such as membranes, proteins, and DNA. Among RNS molecules, peroxynitrite, a product of superoxide anion and nitric oxide, is one of the most prominent one (Liu et al., 2011, 2012; Labbe et al., 2016; Cao et al., 2017; Ramdial et al., 2017). Peroxynitrite can oxidize a wide variety of biomolecules, which, in turn, modulates cell signal transduction pathways, interferes with mitochondrial function, impairs DNA, and finally mediates necrosis and apoptosis in different cell types (Korkmaz et al., 2009). Peroxynitrite participates in the occurrence and development of the varieties of pathological conditions, but data about the effects of peroxynitrite on auditory cells in the cochlea are still very limited. In our previous studies, we found that peroxynitrite induced cytotoxicity in rat SGNs (Liu et al., 2011, 2012) and mouse cochlear HCs (Cao et al., 2017). However, the effect of peroxynitrite on SGNs has not yet been fully elucidated, and it is important to eliminate the excessive peroxynitrite so as to counteract its toxic effect to protect cells from oxidative injuries.

Studies have shown that organisms can activate a series of defense responses, such as improving the activity of antioxidant enzymes in the body and initiating lysosomal degradation pathways, to prevent oxidative damage. Glutathione peroxidase 1 (GPX1) is one of the most abundant members of the GPXs family that protects cell from oxidative damage and maintains the balance of intracellular redox systems (Brigelius-Flohé, 1999). Glutathione peroxidase 1 has been reported for its effect in modulating many pathophysiologic processes in which oxidative stress play a vital role (Lubos et al., 2011). For example, GPX1 might play a protective role in alleviating oxidative stress as a neuromodulator in neurodegenerative disorders (Sharma et al., 2021a), GPX1 is a gatekeeper restraining the oncogenic power of mitochondrial ROS generated by superoxide dismutase 2 (SOD2) (Ekoue et al., 2017), and the upregulation of the GPX1 activity allows the mitochondrial-defective cells to survive oxidative stress and cisplatin treatment (Lu et al., 2012). In the auditory system, a previous study reported that a single nucleotide polymorphism in GPX1 might be associated with the vulnerability to noise-induced hearing loss (NIHL) among the Chinese Han population (Wen et al., 2014). Kil et al. (2007) have declared that GPX1 is the dominant isoform of GPXs family and is highly expressed in HCs, supporting cells, SGNs, stria vascularis, and spiral ligament in the rat cochlea. Additionally, the mice by knockout of GPX1 are more susceptible to NIHL compared with wild type mice (Ohlemiller et al., 2000; McFadden et al., 2001). Nevertheless, the effect of GPX1 on SGNs damage induced by oxidative stress remains unclear.

Ebselen [2-phenyl-1,2-benzoisoselenazol-3(2H)-one] is a synthetic organoselenium radical scavenger compound that has GPX-like activity, and it can directly increase GPX1 activity to mimic the effect of GPX1 overproduction. Generally, ebselen is capable of reducing oxidative stress levels in various cell types potentially through a variety of mechanisms. It has been demonstrated that ebselen possesses otoprotective activity and alleviated NIHL in rat *via* preventing the loss of outer HCs and reduces the acute swelling of the stria vascularis (Kil et al., 2007), it can also attenuate cisplatin-induced ROS generation through Nrf2 activation in auditory cells (Kim et al., 2009). Besides, a phase 2 clinical trial declared the safety and efficacy of ebselen for the prevention of NIHL in human (Kil et al., 2017). Here, we used ebselen, as a GPX1 mimic, and the *gpx1* knockout (*gpx1*^–/–^) mice to investigate the role of GPX1 in SGNs treated with peroxynitrite.

In this study, we first identified the expression change of GPX1 in mouse cochlea SGNs through the *in vitro* peroxynitrite-damaged SGNs model, then we investigated the neuroprotective effect of GPX1 against peroxynitrite-induced SGN damage. Finally, we explored the possible underlying mechanism by which GPX1 was involved in protecting SGNs against peroxynitrite damage. Our findings demonstrated that GPX1 protected against peroxynitrite-induced SGNs damage by inhibiting oxidative stress and apoptosis, at least partially, through inhibiting the activation of nuclear factor-kappa B (NF-κB) pathway.

## Materials and Methods

### Experimental Animals and Genotyping

The C57BL/6 wide type (WT) mice were purchased from the Animal Center of Shandong University (Jinan, China). The *gpx1* knockout mice (*gpx1*^–/–^) in the C57BL/6 background (KOCMP-14775-Gpx1-B6N-VA) were constructed by Cyagen Biosciences Inc. (Suzhou, China). The genotyping for *gpx1*^–/–^ mice with PCR was performed according to the Cyagen Biosciences recommendations. The genotyping primers are listed in Table 1. All animal experiments were performed according to protocols approved by the Animal Care Committee of Shandong University (No. ECAESDUSM 20123011) and were consistent with the National Institute of Health’s Guide for the Care and Use of Laboratory Animals.

**TABLE 1:** PCR primer sequences used in the experiments.

Gene	Forward sequence	Reverse sequence
*Gpx1*- WT	TTACACAATATAAGGGAGCTGTGC	ATACCTGGTGTCCGAACTGATTG
*Gpx1*- Mut	TTACACAATATAAGGGAGCTGTGC	TTAAGGCACTGAGTAGCAGTGTG
*Bax*	CGTGGTTGCCCTCTTCTACT	TTGGATCCAGACAAGCAGCC
*Bcl-2*	TGACTTCTCTCGTCGCTACCG	GTGAAGGGCGTCAGGTGCAG
*p53*	GGTGCTGACGAAGAAGAGGA	AGCCCAACTGTGATGAAGCA
*Bcl-xL*	GGAGCTGGTGGTTGACTTTCT	CCGGAAGAGTTCATTCACTAc
*Gapdh*	AGGTCGGTGTGAACGGATTTG	TGTAGACCATGTAGTTGAGGTCA

### Organotypic Culture of Neonatal Mouse Cochleae Spiral Ganglion Neurons and Drug Treatments

The C57BL/6 WT mice or *gpx1*^–/–^ mice were decapitated at postnatal day (P) 3, and only the middle turn segments of mouse cochleae were collected and cultured for all experiments to keep sampling consistence between groups. The tissue dissection procedure was carried out as described in our previous report (Wang et al., 2019; Liu et al., 2021). Briefly, after cutting off the temporal bones of two sides and removing the cochlear capsule and stria vascularis, the middle turn cochlear explants containing SGNs were then adhered onto 10 mm glass coverslips (Fisher Scientific, PA) pre-coated with CellTak (Corning, 354241). Isolated SGNs explants were cultured in Dulbecco’s Modified Eagle Medium/F12 (DMEM/F12, Gibco, 11330032) supplemented with 10% fetal bovine serum (FBS, Gibco, 10099133C) and ampicillin (50 mg/ml, Sigma, A5354) overnight at 37°C in a 5% CO_2_ atmosphere.

On the following day, samples were changed into fresh culture media containing peroxynitrite (Cayman, 81565) alone for 24 or 48 h, or with the following drugs for 48 h as indicated in the text: ebselen (30 μM, Sigma-Aldrich, E3520); NAC (2 mM, Sigma-Aldrich, A7250); BAY 11-7082 (10 μM, Med Chem Express, HY-13453). After incubation, samples were used in the immunostaining or other assays.

### Cryosection

Cochleae from P3, P14, and P30 C57BL/6 WT mice were dissected out and fixed with 4% paraformaldehyde (PFA) in PBS at 4°C overnight. Tissues were then incubated in 10, 20, and 30% sucrose in 1 × PBS, embedded in OCT compound (Tissue-Tek, Sakura Finetek, 4583), snap frozen on dry ice, and then stored frozen at −80°C. Frozen sections were cut into 7 μm using a cryostat (Leica CM 1850; Leica, Germany).

### Immunostaining

After organotypic culture or cryosection, the cochlear explants or tissue sections were fixed with 4% PFA, permeabilized with 1% TritonX-100 in PBS, and blocked by incubation in PBT-1 solution (0.1% Triton X-100, 8% donkey serum, 1% bovine serum albumin, and 0.02% sodium azide in PBS) at room temperature for 1 h. The samples were then incubated with the following primary antibodies: anti-Tuj 1 (1:1,000 dilution; Neuromics, MO15013), anti-GPX1 (1:500 dilution, GeneTex, GTX116040), anti-C-caspase 3 (1:400 dilution, Cell Signaling Technology, 9664), anti-NeuN (1:500, Cell Signaling Technology,12943), anti-4-HNE (1:500 dilution, Abcam, ab48506, United States), or anti-NF-κB p65 (1:500 dilution, Cell Signaling Technology, 6956) diluted in the blocking solution at 4°C overnight. The next day, samples were incubated with FITC-conjugated, TRITC-conjugated, or Cy5-conjugated (1:1000, Invitrogen, United States) secondary antibody along with 4′,6-diamidino-2-phenylindole (DAPI) (1:1000, Sigma-Aldrich, United States) at room temperature for 1 h. Then, coverslips were mounted and the samples were observed under a laser scanning confocal microscope (Leica SP8; Leica, Germany).

### Western Blot

After the drug treatment, the proteins from the cultured SGNs were extracted with radioimmunoprecipitation assay buffer (RIPA) buffer (Protein Biotechnology, China). The mixture was centrifuged at 4°C and 12,000 × *g* in a refrigerated centrifuge and the supernatant was collected. A total of 30 μg of each protein sample was denatured and separated by 12% sodium dodecyl sulfate–polyacrylamide gel electrophoresis (SDS-PAGE) gels. The primary antibodies were anti-GPX1 (1:1,000 dilution, GeneTex, GTX116040), anti-C-caspase 3 (1:500 dilution, Cell Signaling Technology, 9664), anti-4-HNE (1:1000, Abcam, ab46545), anti-phosphorylated (p)-NFκB p65 (1:1,000 dilution, Cell Signaling Technology, 3033), and anti-β-actin (1:2,000 dilution, ZSGB-BIO, TA-09). The protein signals were detected using an ECL kit (Millipore, United States) and analyzed by Image J software. The relative optical density ratio was calculated by comparison to β-actin.

### Real Time-Polymerase Chain Reaction

After drug treatment for 48 h, total RNA of different groups was isolated from middle turn cochlear explants using TRIzol reagent (Life Technologies, 15596026) following manufacturer’s instructions. The cDNA was synthesized from each RNA sample by reverse transcription using the Revert Aid First Strand cDNA Synthesis Kit (Thermo Fisher Scientific, K1622). A SYBR Premix Ex Taq (TaKaRa Bio, RR420A) was used to quantify the mRNA levels of related genes. *Gapdh* was amplified as the housekeeping gene. All data were analyzed using an Eppendorf Realplex 2 and the relative expression levels were calculated using the 2^–Δ^ ^Δ^ *^CT^* method. PCR primers for the genes were listed in Table 1.

### Spiral Ganglion Neuron Counting

The counting of SGNs was performed according to our previous researches (Wang et al., 2019; Liu et al., 2021). Briefly, SGNs were immunolabeled with the Tuj 1 antibody that specifically labels both SGN bodies and neurites. The images of cultured middle turn cochlear explants were taken using a Leica confocal fluorescence microscope. Spiral ganglion neurons in which the nucleus comprised 40% of the soma area were counted using the Image J software and the total number of SGNs in each spiral ganglion explant was obtained by adding the SGN counts in all consecutive sections. The density of SGNs was then calculated per unit area (0.01 mm^2^).

### Statistical Analysis

All experiments were repeated at least three times and the data were presented as mean ± SEM. Two-tailed, unpaired Student’s *t*-tests were used to determine statistical significance in comparisons between two groups. When comparing more than two groups, data were statistically analyzed by one-way ANOVA followed by a Dunnett multiple comparisons test. A value of *p* < 0.05 was considered to indicate a statistically significant result. Scale bars and *n* values are defined in the respective figures and legends and *n* represents the number of independent cochlear samples from each sub-group.

## Results

### Glutathione Peroxidase 1 Expression Is Decreased in Spiral Ganglion Neurons After Peroxynitrite Injury

**FIGURE 1:**
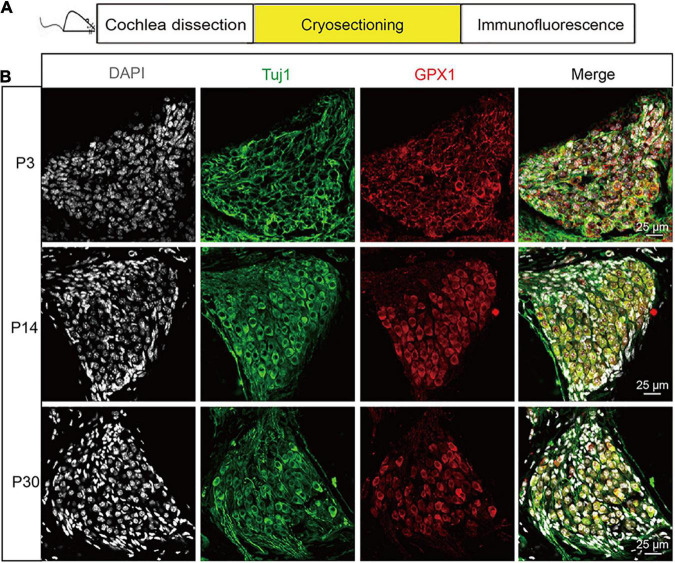
The expression of GPX1 in postnatal cochlear spiral ganglion neurons (SGNs) with mice at different ages. **(A)** Immunofluorescence staining was performed on the frozen cochlear cryosection of P3, P14, and P30 C57BL/6 mouse cochlea. **(B)** Immunostaining images of the middle turn cochlea showed that robust GPX1 labeling was observed in cochlear SGNs of P3, P14, and P30 mice, and it appeared that the expression of GPX1 was mainly in the cytoplasm but not in the nuclei of SGNs. Scale bar = 25 μm.

First, we characterized the expression of GPX1 in postnatal cochlear SGNs with mice at different ages. Immunofluorescence staining was performed on the frozen cochlear cryosections of P3, P14, and P30 C57BL/6 mouse cochleae (Figure 1A), and Tuj 1, a neuron-specific marker which specifically labels both the cytoplasm and neurite of SGN, was used to mark SGN. As the expression pattern of GPX1 was the same in all three turns of cochlea SGNs, the middle turn was presented as the representative sample in Figure 1B. The results showed that robust GPX1 labeling was observed in cochlear SGNs of P3, P14, and P30 mice, and it appeared that the expression of GPX1 was mainly in the cytoplasm but not in the nuclei of SGNs (Figure 1B).

**FIGURE 2:**
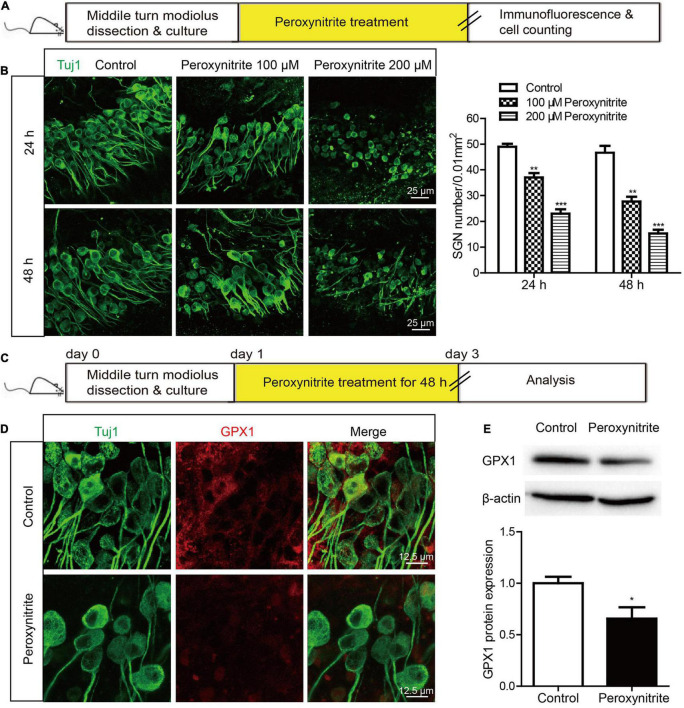
The expression of GPX1 is decreased in cochlear SGNs after peroxynitrite injury. **(A)** The cultured SGNs from WT C57BL/6 mice were treated with different concentrations (100 or 200 μM) of peroxynitrite for 24 or 48 h, respectively. **(B)** Immunostaining images showed that the SGN morphology was disrupted, cells arrangement was disordered, and nerve fibers were disordered or lost after peroxynitrite administration. Quantitative analysis verified that the numbers of survived SGNs were reduced significantly in peroxynitrite-treated groups in a dose- and time-dependent manner compared with the control group. Scale bar = 25 μm. **(C)** The cultured SGNs were exposed to 100 μM peroxynitrite for 48 h. **(D)** Immunostaining illustrated that the fluorescence intensity of GPX1 in peroxynitrite group was significantly reduced compared with control group. Scale bar = 12.5 μm. **(E)** Western blot showed that the protein level of GPX1 was significantly decreased in peroxynitrite group compared with the control group. *p < 0.05, **p < 0.01, and ***p < 0.001, *n* = 3 for each group.

Next, to determine the neurotoxic effect of peroxynitrite on cultured SGNs, the cultured SGNs were treated with different concentrations (100 or 200 μM) of peroxynitrite for 24 or 48 h, respectively (Figure 2A). As shown in Figure 2B, after the administration of peroxynitrite, the SGN morphology was disrupted and pyknotic, cells arrangement was disordered, nerve fibers were disordered, broken, or lost. Quantitative analysis showed that the numbers of survived SGNs were reduced significantly in peroxynitrite-treated groups in a dose- and time-dependent manner compared with the control group (Figure 2B), that is, the higher concentration of peroxynitrite and the longer processing time induced more SGNs loss. In detail, treatment with 100 μM peroxynitrite for 24 h induced minor SGN loss (75.4 ± 2.49% SGNs survived after treatment) and the higher concentration of 200 μM peroxynitrite treatment for 48 h caused approximately only 32.7 ± 1.84% SGNs survival compared with controls. The treatment of 100 μM peroxynitrite exposure for 48 h resulted in an obvious but moderate SGN loss, as there were 59.3 ± 3.71% SGNs left compared with the control group (Figure 2B), and it was selected as the peroxynitrite treatment condition for all subsequent experiments.

Then, the expression change of GPX1 in SGNs was analyzed after peroxynitrite damage. The cultured SGNs were exposed to 100 μM peroxynitrite for 48 h (Figure 2C) and immunostaining illustrated that the fluorescence intensity of GPX1 in the peroxynitrite group was significantly reduced compared with that of the control group (Figure 2D). Western blot showed that the protein level of GPX1 was significantly decreased in peroxynitrite group compared with the control group (Figure 2E). Together, these results indicated that the administration of peroxynitrite caused a reduced expression of GPX1 in cochlear SGNs, thus suggesting that GPX1 might play a role in peroxynitrite-induced SGN damage.

### Glutathione Peroxidase 1 Promotes the Survival of Spiral Ganglion Neurons After Peroxynitrite Damage

**FIGURE 3:**
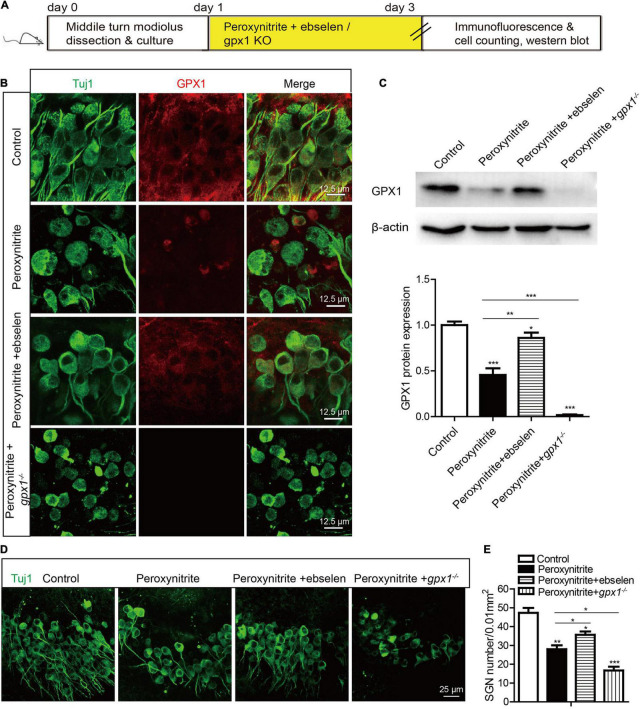
Glutathione peroxidase promotes the survival of SGNs after peroxynitrite damage. The cultured SGNs from P3 WT mice were incubated with 100 μM peroxynitrite for 48 h with or without pretreatment of 30 μM ebselen for 1 h, or the SGNs from *gpx1*^–/–^ mice were cultured and exposed to 100 μM peroxynitrite for 48 h. **(A–C)** Immunofluorescence staining and western blot results showed that the protein expressions of GPX1 were significantly increased in peroxynitrite + ebselen group compared with the peroxynitrite-only group, while it was absent in the peroxynitrite + *gpx1*^–/–^ group. Scale bar = 12.5 μm. **(D,E)** Immunostaining and cell counting showed that the number of surviving SGNs was significantly increased after pretreatment with ebselen compared with the peroxynitrite-only group, while remarkably less survived SGNs were detected in *gpx1*^–/–^ mice group compared with WT mice after peroxynitrite treatment. Scale bar = 25 μm. *p < 0.05, **p < 0.01, ***p < 0.001, and *n* = 3 for each group.

To clarify the role of GPX1 in the process of SGN injury caused by peroxynitrite, we performed experiments by increasing or inhibiting the expression of GPX1 in SGNs with the GPX1 mimic ebselen or using the *gpx1*^–/–^ mouse model. Immunofluorescence staining and western blot results verified that GPX1 expression was absent in the cochlear SGNs of *gpx1*^–/–^ mice (Supplementary Figures 1A,C), and there was no significant difference in SGNs number between the *gpx1*^–/–^ mice and WT mice (Supplementary Figure 1B), which indicated that the absence of GPX1 in normal SGNs would not affect the survival of SGNs. Next, we examined the effect of regulating GPX1 expression by ebselen or GPX1 deficiency on peroxynitrite-induced SGNs loss. The cultured SGNs from P3 WT mice were incubated with 100 μM peroxynitrite for 48 h with or without the pretreatment of 30 μM ebselen for 1 h, or the SGNs from *gpx1*^–/–^ mice were cultured and exposed to 100 μM peroxynitrite for 48 h. The dose of ebselen was chosen according to our published study (Liu et al., 2021), which shows that 30 μM ebselen treatment protects against cisplatin-induced oxidative stress in SGNs. As illustrated in Figure 3, the protein expressions of GPX1 were significantly increased in peroxynitrite + ebselen group compared with the peroxynitrite-only group, while it was absent in the peroxynitrite + *gpx1*^–/–^ group (Figures 3A–C). Immunostaining and cell counting results showed that the number of surviving SGNs was significantly increased after the pretreatment with ebselen compared with the peroxynitrite-only group, while remarkably less survived SGNs were detected in *gpx1*^–/–^ mice group compared with WT mice after peroxynitrite treatment (Figures 3D,E). These results suggested that the upregulation of GPX1 promotes, while the deficiency of GPX1 suppresses, the survival of SGNs after peroxynitrite injury.

### Glutathione Peroxidase 1 Inhibits Apoptosis of Spiral Ganglion Neurons After Peroxynitrite Injury

**FIGURE 4:**
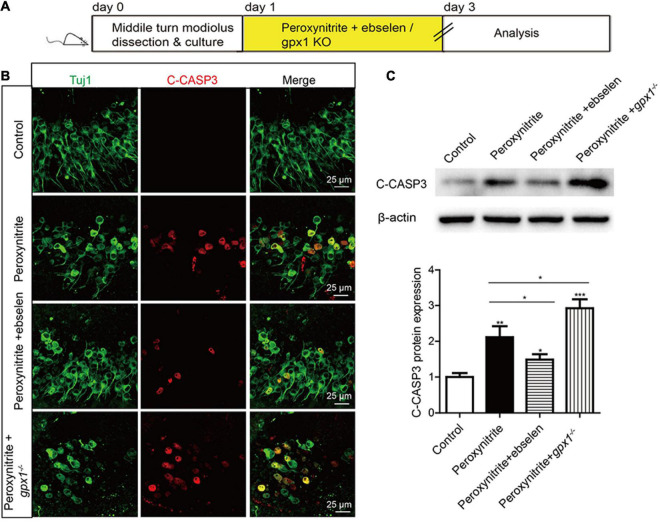
Glutathione peroxidase inhibits the apoptosis of SGNs after peroxynitrite injury. **(A)** The cultured SGNs from P3 WT mice were incubated with 100 μM peroxynitrite for 48 h with or without the pretreatment of 30 μM ebselen for 1 h, or the SGNs from *gpx1*^–/–^ mice were cultured and exposed to 100 μM peroxynitrite for 48 h. **(B)** Immunostaining results showed that distinct C-CASP3-positive SGNs were observed in the peroxynitrite group, but not in the control group. The number of C-CASP3-positive SGNs was downregulated in the presence of ebselen, while it was further increased in *gpx1*^–/–^ mice compared with the peroxynitrite-only group. Scale bar = 25 μm. **(C)** The western blot analysis of C-CASP3 protein verified that the expression level of C-CASP3 was significantly reduced in the peroxynitrite + ebselen group, while it was significantly increased in the peroxynitrite + *gpx1*^–/–^ group compared with the peroxynitrite-only group. *p < 0.05, **p < 0.01, and ***p < 0.001, *n* = 3 for each group.

The role of GPX1 in regulating the peroxynitrite-induced apoptosis of cochlear SGNs was investigated by immunostaining and western blot. After the drug treatment (Figure 4A), cleaved-Caspase3 (C-CASP3) immunostaining results showed that distinct C-CASP3-positive SGNs were observed in peroxynitrite group, but not in the control group. The number of C-CASP3-positive SGNs was downregulated in the presence of ebselen, while it was further increased in *gpx1*^–/–^ mice compared with the peroxynitrite-only group (Figure 4B). Western blot analysis of C-CASP3 protein was consistent with the above results, i.e., the expression level of C-CASP3 was significantly reduced in the peroxynitrite + ebselen group, while it was significantly increased in the peroxynitrite + *gpx1*^–/–^ group compared with the peroxynitrite-only group (Figure 4C). Therefore, these results revealed that the Caspase 3-mediated apoptosis was inhibited by ebselen and was aggravated by the lack of GPX1 in SGNs after peroxynitrite injury.

### Glutathione Peroxidase 1 Attenuates Peroxynitrite-Induced Oxidative Stress in Spiral Ganglion Neurons

**FIGURE 5:**
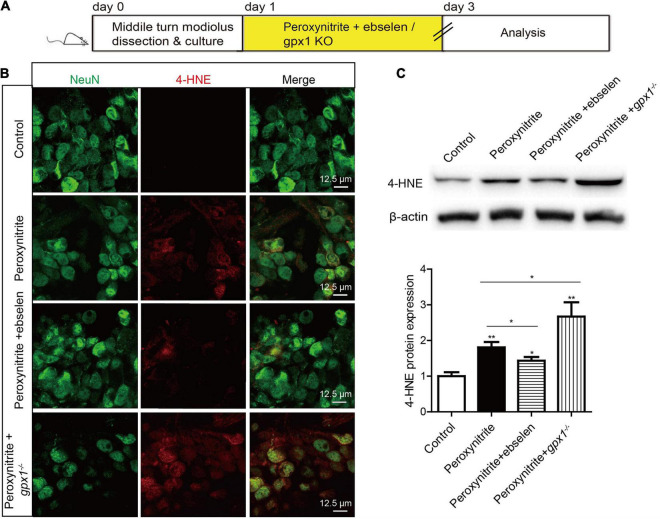
Glutathione peroxidase attenuates peroxynitrite-induced oxidative stress in SGNs. **(A)** The cultured SGNs from P3 WT mice were incubated with 100 μM peroxynitrite for 48 h with or without the pretreatment of 30 μM ebselen for 1 h, or the SGNs from *gpx1*^–/–^ mice were exposed to 100 μM peroxynitrite for 48 h. **(B,C)**. Immunostaining and western blot results showed that obvious increased protein expression of 4-HNE was found in peroxynitrite treated SGNs, while it was significantly decreased in the peroxynitrite + ebselen group but further increased in the peroxynitrite + *gpx1*^–/–^ mice compared with the peroxynitrite-only group. Scale bar = 12.5 μm. *p < 0.05, and **p < 0.01, *n* = 3 for each group.

Glutathione peroxidase 1 is known as a major intracellular peroxide-scavenging enzyme to eliminate the damage of ROS to cells, proteins, and liposomes through the reduction of glutathione. In this study, we also investigated the relationship between GPX1 and ROS generation in peroxynitrite damaged SGNs. The cultured SGN explants from WT or *gpx1*^–/–^ mice were exposed to peroxynitrite with or without ebselen pretreatment, and the expression changes of 4-HNE were examined to evaluate the oxidative stress level in SGNs (Figure 5A). The results of immunostaining and western blot showed that an obvious increased protein expression of 4-HNE was found in peroxynitrite treated SGNs, while it was significantly decreased in peroxynitrite + ebselen group but further increased in peroxynitrite + *gpx1*^–/–^mice compared with the peroxynitrite-only group (Figures 5B,C).

### Antioxidant Treatment Rescues the Aggravated Spiral Ganglion Neurons Loss and Apoptosis Induced by Glutathione Peroxidase 1 Deficiency After Peroxynitrite Injury

**FIGURE 6:**
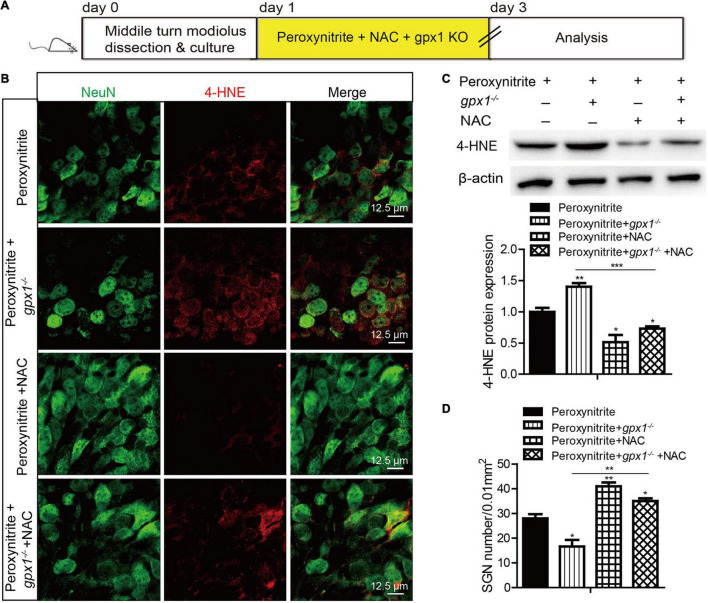
Antioxidant treatment rescues the aggravated SGNs loss and ROS accumulation induced by GPX1 deficiency after peroxynitrite injury. **(A)** The cultured SGNs from WT or *gpx1*^–/–^ mice were treated with peroxynitrite and 2 mM NAC for 48 h. **(B,C)** Immunostaining results illustrated that the immunofluorescence of 4-HNE was weaker in the peroxynitrite + NAC group compared with the peroxynitrite-only group, and it was also lower in the peroxynitrite + *gpx1*^–/–^ + NAC group compared with the peroxynitrite + *gpx1*^–/–^ group. Western blot results verified that the protein expression level of 4-HNE was consistent with the immunostaining results. Scale bar = 12.5 μm. **(D)** Cell counting and statistical analysis showed that the NAC treatment largely increased the number of surviving SGNs in the peroxynitrite + N-acetylcysteine (NAC) group compared with the peroxynitrite-only group, as well as in the peroxynitrite + *gpx1*^–/–^ + NAC group compared with the peroxynitrite + *gpx1*^–/–^ group. *p < 0.05, **p < 0.01, and ***p < 0.001, *n* = 3 for each group.

**FIGURE 7:**
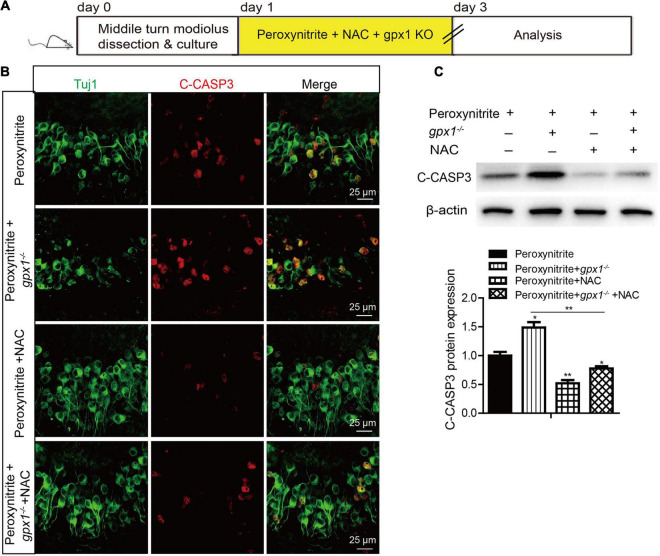
N-acetylcysteine treatment reduced the apoptosis of SGNs in the lacking of GPX1 expression after peroxynitrite injury. **(A)** The cultured SGNs from WT or *gpx1*^–/–^ mice were treated with peroxynitrite and 2 mM NAC for 48 h. **(B)** Immunostaining results illustrated that there was much less C-CASP3-positive SGNs in the peroxynitrite + NAC group compared with the peroxynitrite-only group, as well as in the peroxynitrite + *gpx1*^–/–^ + NAC group compared with the peroxynitrite + *gpx1*^–/–^ group. Scale bar = 12.5 μm. **(C)** Western blot results verified that the protein expression level of C-CASP3 was weaker in the peroxynitrite + NAC group compared with the peroxynitrite-only group, and it was also lower in the peroxynitrite + *gpx1*^–/–^ + NAC group compared with the peroxynitrite + *gpx1*^–/–^ group. *p < 0.05, and **p < 0.01, *n* = 3 for each group.

To explore whether GPX1 plays a protective role in peroxynitrite-induced SGNs damage by reducing intracellular ROS, we conducted a rescue experiment by using the antioxidant N-acetylcysteine (NAC). The cultured SGN explants from WT or *gpx1*^–/–^ mice were treated with peroxynitrite and 2 mM NAC for 48 h (Figures 6A, 7A). The dose of NAC was chosen according to our published studies (Liu et al., 2019b,^2021^), which show that 2 mM NAC treatment successfully rescues the SGN loss from cisplatin damage. Immunostaining results illustrated that the immunofluorescence of 4-HNE was weaker in the peroxynitrite + NAC group compared with the peroxynitrite-only group, and it was also lower in the peroxynitrite + *gpx1*^–/–^ + NAC group compared with the peroxynitrite + *gpx1*^–/–^ group (Figure 6B). Western blot and statistical analysis of the protein expression level of 4-HNE were consistent with the immunostaining results (Figure 6C). Correspondingly, we found that the NAC treatment largely increased the number of surviving SGNs in the peroxynitrite + NAC group compared with the peroxynitrite-only group, as well as in the peroxynitrite + *gpx1*^–/–^ + NAC group compared with the peroxynitrite + *gpx1*^–/–^ group (Figure 6D). Furthermore, the apoptosis of SGNs detected by C-CASP3 immunostaining and western blot showed that there was much less C-CASP3-positive SGNs and remarkable reduction of the C-CASP3 protein levels in the NAC co-treated groups compared with the peroxynitrite-only groups, and it was also lower in the peroxynitrite + *gpx1*^–/–^ + NAC group compared with the peroxynitrite + *gpx1*^–/–^ group (Figures 7B,C). Collectively, these findings suggest that the antioxidant NAC treatment successfully rescued the exacerbated ROS generation, SGNs loss, and apoptosis caused by GPX1-deficiency after peroxynitrite damage.

### Glutathione Peroxidase 1 Inhibits the NF-κB Pathway to Protect Against Peroxynitrite-Induced Spiral Ganglion Neurons Damage

**FIGURE 8:**
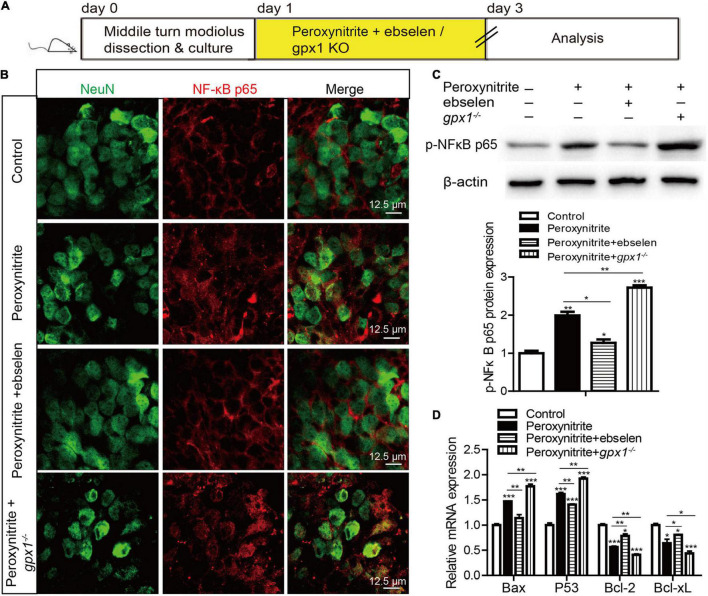
The activated nuclear factor-kappa B (NF-κB) pathway is inhibited by GPX1 in SGNs after peroxynitrite damage. **(A)** The cultured SGNs from P3 WT mice were incubated with 100 μM peroxynitrite for 48 h with or without the pretreatment of 30 μM ebselen for 1 h, or the SGNs from *gpx1*^–/–^ mice were exposed to 100 μM peroxynitrite for 48 h. **(B)** The anti-NeuN antibody was used to label the nucleus of SGN, and the immunostaining results showed that the fluorescence of NF-κB p65 was mainly detected in the cytoplasm of SGNs in the control group. Peroxynitrite induced obvious nuclear distribution of NF-κB p65 in SGNs and pretreatment with ebselen significantly reduced it, whereas the lack of GPX1 intensified the nuclear fluorescence of NF-κB p65 in SGNs of the peroxynitrite + *gpx1*^–/–^ group. Scale bar = 12.5 μm. **(C)** Western blot results verified that the expression of the p-NF-kB p65 protein was upregulated in peroxynitrite-exposed SGNs compared with the untreated controls, and the pretreatment with ebselen significantly reduced it while the lack of GPX1 increased it. **(D)** Real-time PCR (RT-PCR) results revealed that peroxynitrite treatment caused significant increases in the microRNA (mRNA) expression of proapoptotic genes *Bax* and *P53*, and decreased the expression of antiapoptotic genes *Bcl2*, *Bcl-xL* compared with controls. The lower mRNA expression levels of *Bax* and *P53* and the higher expression levels of *Bcl2* and *Bcl-xL* were found in the peroxynitrite + ebselen group compared with the peroxynitrite-only group, while the lack of GPX1 in SGNs from *gpx1*^–/–^ mice led to an opposite pattern. *p < 0.05, **p < 0.01, and ***p < 0.001, *n* = 3 for each group.

The transcription factor NF-κB plays a vital role in cellular death and survival under oxidative stress conditions (Li and Karin, 1999), and the activation of NF-κB can promote cell death (Schneider et al., 1999; Zhang et al., 2005). Recently, the correlation between GPX1 and NF-κB pathway has been declared (Koeberle et al., 2020; Sharma et al., 2021b). Therefore, to identify the role of NF-κB and the regulative mechanism between GPX1 and NF-κB in SGNs damage induced by peroxynitrite, we measured the NF-κB pathway in SGNs after peroxynitrite administration with GPX1 upregulation or deficiency (Figure 8A). As illustrated in Figure 8B, the anti-NeuN antibody was used to label the nucleus of SGN and the fluorescence of NF-κB p65 was mainly detected in the cytoplasm of SGNs in control group (Figure 8B). Peroxynitrite induced obvious nuclear distribution of NF-κB p65 in SGNs and pretreatment with ebselen significantly reduced it, whereas the lack of GPX1 intensified the nuclear fluorescence of NF-κB p65 in SGNs of peroxynitrite + *gpx1*^–/–^ group (Figure 8B). Furthermore, we found that the expression of the p-NF-κB p65 protein was upregulated in peroxynitrite-exposed SGNs compared with the untreated controls, and the pretreatment with ebselen significantly reduced it while the lack of GPX1 increased it (Figure 8C). Besides, to further confirm peroxynitrite-mediated activation of the NF-κB pathway, we also measured the mRNA expression levels of known NF-κB p65 target genes *Bax*, *P53*, *Bcl2*, and *Bcl-xL*. Real time-PCR (RT-PCR) results revealed that peroxynitrite treatment caused significant increases in the mRNA expression of proapoptotic genes *Bax* and *P53*, and decreases in the mRNA expression of antiapoptotic genes *Bcl2* and *Bcl-xL* compared with controls (Figure 8D). In addition, the lower mRNA expression levels of *Bax* and *P53* and the higher expression levels of *Bcl2* and *Bcl-xL* were detected in the peroxynitrite + ebselen group compared with the peroxynitrite-only group, while the lack of GPX1 in SGNs from *gpx1*^–/–^ mice led to an opposite pattern (Figure 8D). These data, taken together, suggest that the NF-κB pathway is activated in SGNs after peroxynitrite treatment and GPX1 can partially block the nuclear translocation of NF-κB/p65 and inhibit the activation of the NF-κB signaling.

**FIGURE 9:**
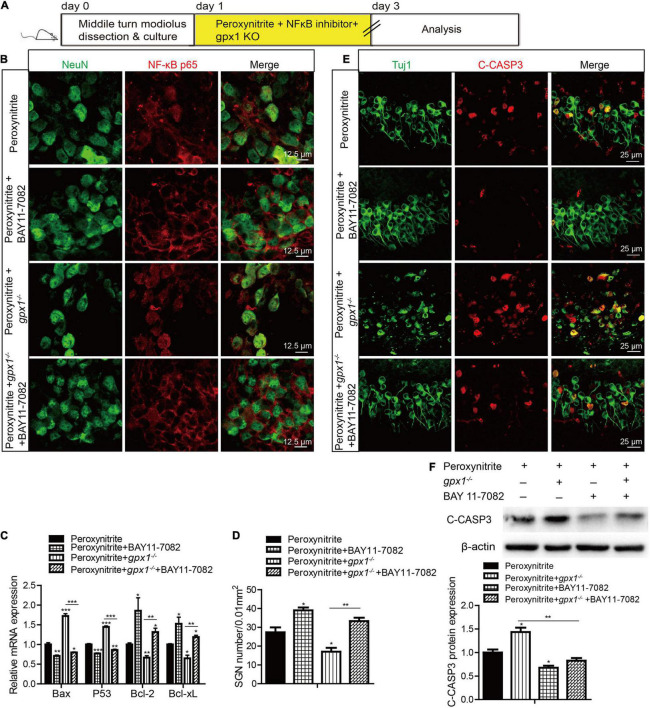
Glutathione peroxidase inhibits the NF-κB pathway to protect against peroxynitrite-induced SGNs damage. **(A)** The cultured SGN explants from WT or *gpx1*^–^*^/^*^–^ mice were treated with peroxynitrite and 10 μM BAY 11-7082 for 48 h. **(B,C)** Immunostaining and RT-PCR results showed that BAY 11-7082 effectively inhibited the nuclear translocation of NF-κB p65 and the mRNA expression of *Bax* and *P53*, but increased the mRNA expression of *Bcl2* and *Bcl-xL*, in the peroxynitrite + BAY 11-7082 group compared with the peroxynitrite-only group, as well as in the peroxynitrite + *gpx1*^–/–^ + BAY 11-7082 group compared with the peroxynitrite + *gpx1*^–/–^ group. Scale bar = 12.5 μm. **(D–F)** Immunostaining, cell counting, and western blot results showed that the inhibition of NF-κB by BAY 11-7082 significantly increased the survived SGN numbers, while decreased the C-CASP3 positive SGNs numbers and the C-CASP3 expression in the peroxynitrite + BAY 11-7082 group compared with the peroxynitrite group. Moreover, BAY 11-7082 treatment also increased the number of SGNs, whereas reduced the C-CASP3 expression in the peroxynitrite + *gpx1*^–/–^ + BAY 11-7082 group vs. the peroxynitrite + *gpx1*^–/–^ group. Scale bar = 25 μm. *p < 0.05, **p < 0.01, and ***p < 0.001, *n* = 3 for each group.

Next, we used a highly potent NF-κB inhibitor, BAY 11-7082, to further verify the role of NF-κB pathway in SGNs damage induced by peroxynitrite (Figure 9). The cultured SGN explants from WT or *gpx1*^–/–^ mice were treated with peroxynitrite and 10 μM BAY 11-7082 for 48 h. The dose of BAY11-7082 was chosen based on our preliminary results of dose response, which showed that 10 μM BAY11-7082 significantly increased the number of surviving SGNs after cisplatin damage *in vitro* (Supplementary Figure 2). Our results showed that 10 μM BAY 11-7082 effectively inhibited the nuclear translocation of NF-κB p65 and the mRNA expression of *Bax* and *P53*, but increased the mRNA expression of *Bcl2* and *Bcl-xL* in the peroxynitrite + BAY 11-7082 group compared with the peroxynitrite-only group, as well as in the peroxynitrite + *gpx1*^–/–^ + BAY 11-7082 group compared with the peroxynitrite + *gpx1*^–/–^ group (Figures 9A–C). Correspondingly, the inhibition of NF-κB by BAY 11-7082 significantly increased the survived SGN numbers, while decreased the C-CASP3 positive SGN numbers and the C-CASP3 expression in peroxynitrite + BAY 11-7082 group compared with the peroxynitrite group (Figures 9D–F). More importantly, BAY 11-7082 treatment also rescued the aggravated SGN loss and apoptosis induced by the deficiency of GPX1 after peroxynitrite injury, as the number of SGNs was added, whereas the C-CASP3 expression was reduced in the peroxynitrite + *gpx1*^–/–^ + BAY 11-7082 group vs. the peroxynitrite + *gpx1*^–/–^ group (Figures 9D–F). Therefore, these results indicate that the inhibition of NF-κB pathway contributes to promote SGNs survived from peroxynitrite injury, and that GPX1 protects SGNs against peroxynitrite-induced damage, at least in part, *via* blocking the NF-κB pathway activation.

## Discussion

Glutathione peroxidase is a crucial antioxidant enzyme that participate in restraining the harmful accumulation of intracellular hydrogen peroxide and is more effective than catalase at eliminating intracellular peroxides under various physiological conditions (Antunes et al., 2002; Lubos et al., 2011). Here, for the first time, we reported that the expression of GPX1, which was observed robustly distributed in SGNs cytoplasm, was significantly reduced after peroxynitrite damage. Our finding is consistent with previous studies indicated that a decrease of GPX1 activity was found in mouse cochlea HCs and stria vascular after noise induced hearing loss (McFadden et al., 2001; Kil et al., 2007), and suggested that the antioxidant enzyme GPX1 might play a role in the oxidative injury of SGNs induced by peroxynitrite.

In the present study, the role of GPX1 in SGNs treated with peroxynitrite was explored *via* using ebselen and GPX1 deficient mice. Ebselen was found to increase the expression of GPX1 in SGNs after peroxynitrite damage, which is consistent with the reports that ebselen can increase the expression of GPX1 in cochlea after electrical stimulation (Liang et al., 2019) and in stria vascularis after noise exposure (Kil et al., 2007). Ebselen effectively promotes SGN survival and prevents SGN apoptosis after peroxynitrite injury by reducing SGNs oxidative stress, while the absence of GPX1 in *gpx1*^–/–^ mice leads to the aggravated SGN injury (cell loss and apoptosis) induced by peroxynitrite, although it might cause no effect on SGN development as SGNs presented normal morphology and cell number in *gpx1*-deficient mice. These results suggest a neuroprotective effect of GPX1 against the peroxynitrite-induced SGN damage. It is important to note that ebselen mimics the activities of all the selenium-dependent mammalian GPXs, not only to GPX1, and has other effects on redox status. Thus, its protective effects overlap those of GPX1. Other types of GPXs have been shown to be expressed in the cochlea, for instance, GPX2 protein is greatly increased in chicken utricle HC expression during HC differentiation (Zhu et al., 2019) and cisplatin injury leads to the decreased GPX2 expression in HEI-OC1 cells (Youn et al., 2017; Jo et al., 2019). It has been reported that GPX3 and GPX4 labeling are absent in the spiral ganglia, while GPX1 is the major isoform of GPXs family that highly expressed in cochlear SGNs in adult rat (Kil et al., 2007). In this study, we also examined the expression of GPX2 and GPX4 in mouse SGNs, and relative low expressions of GPX2 and GPX4 were found in mouse SGNs (Supplementary Figure 3). Moreover, ebselen increased the expression of GPX2 and GPX4 in SGNs after peroxynitrite injury (Supplementary Figure 3), which is consistent with previous studies that ebselen can increase the expression of GPX2 or GPX4 in rat stomach (Kumar et al., 2010), gastric cancer cells (Xu et al., 2018), or cochlea (Liang et al., 2019). GPX2 and GPX4 serve a distinct function in antioxidant and cellular protection in some tissues that complements that of GPX1 and experimental evidence hints that the expression of GPX2 and GPX4 is less sensitive to variations in selenium levels compared with GPX1 (Lubos et al., 2011). However, to date, studies about the specific function of GPX2 or GPX4 in SGN are still lacking. Our preliminary data suggest that they might also play a part in SGNs against oxidative stress, which is worthy to be investigated in future studies.

With regard to how GPX1 influences the apoptotic outcome, the pieces of evidence indicate that GPX1 may influence several steps in apoptotic cascades *via* regulating oxidant accumulation. For example, one study revealed that an overexpression of GPX1 inhibited the nucleus-translocation of apoptosis-inducing factor (AIF) from mitochondria in neuronal cells after ischemia-induced apoptosis (Zemlyak et al., 2009), another analysis showed that GPX1 decreased the ratio of Bax:Bcl-2 to create a more antiapoptotic environment in human endothelial cells (Faucher et al., 2005). It is known that the intrinsic pathway of apoptosis involves the mitochondria release of pro-apoptotic factors, such as AIF or cytochrome c, and these processes can be activated by ROS. Therefore, it is possible that GPX1, as an essential antioxidant enzyme, attenuates AIF release and enhances the expression of Bcl-2 to inhibit cell apoptosis, by reducing ROS level. In the current study, our results suggest that GPX1 protects against peroxynitrite-induced SGNs damage by suppressing ROS accumulation in SGNs. Interestingly, we have reported that intranuclear localization of AIF involves in the peroxynitrite-induced apoptosis of SGNs (Liu et al., 2012), whether GPX1 regulates the intranuclear localization of mitochondrial AIF to alleviate apoptosis in SGNs after peroxynitrite injury deserved further exploration.

Finally, we explored the possible signaling pathways by which GPX1 was involved in protecting SGNs against peroxynitrite-induced injury. It has been shown that GPX1 can alter the activation of NF-κB (Li et al., 2001), modulate Akt pathways (Handy et al., 2009) to affect cellular proliferation and survival, and modify the ratio of Bax:Bcl-2 to create a more antiapoptotic environment (Faucher et al., 2005). NF-κB is known to be a redox-sensitive transcription factor in several cell types and involved in cellular death and survival under oxidative stress conditions (Li and Karin, 1999; Song et al., 2021). In this study, we found that the NF-κB pathway is activated in SGNs after peroxynitrite treatment and that the inhibition of NF-κB pathway contributes to promote SGNs survived from peroxynitrite injury. More importantly, we discovered that GPX1 can partially block the nuclear translocation of NF-κB p65 and inhibit the activation of the NF-κB signaling to protect SGNs against peroxynitrite damage. This observation is interesting that NF-κB activation is considered anti-apoptotic and pro-survival, partially due to augment the expression of IAPs and thus attenuating the activation of caspase. For instance, a study reported that the NF-κB signaling pathway is activated in noise-exposed cochleae to protect against inducible nitric oxide synthase-triggered oxidative stress and apoptosis (Tamura et al., 2016). We speculate the controversial effect of NF-κB pathway activation on inner ear cells, i.e., protecting or damaging cells, might rely on the cell and stimuli types. Furthermore, one possible explanation for the pro-apoptotic effect of NF-κB activation is that in the context of GPX1 deficiency, excess accumulation of cellular ROS might alter NF-κB responses. Evidence showed that excess intracellular hydrogen peroxide regulates the expression of various NF-κB component proteins, and the alterations in the composition or quantity of the NF-κB dimer can change down-stream target gene expression, and conduce to the upregulation of pro-inflammatory genes and a pro-apoptotic environment (Li et al., 2001; Oliveira-Marques et al., 2009; Lubos et al., 2011).

In summary, we investigated the role of GPX1 in protecting SGNs against oxidative stress by upregulating or inhibiting the expression of GPX1 in SGNs *via* the GPX1 mimic ebselen or the *gpx1* knockout mouse model, and further determined the mechanistic details behind the neuroprotective effect of GPX1 in SGNs against peroxynitrite injury. We found that the ebselen could significantly promote SGN survival, decrease SGN apoptosis, and reduce intracellular ROS levels after peroxynitrite exposure *in vitro*, while the deficiency of GPX1 led to opposite patterns of the above effects. Moreover, we showed that the protective mechanism of GPX1 involves inhibiting the activation of NF-κB pathway in SGNs exposed to peroxynitrite. These findings suggest that GPX1 might serve as a novel target for the prevention of oxidative stress-induced SGNs damage and hearing loss.

## Data Availability Statement

The original contributions presented in the study are included in the article/Supplementary Material, further inquiries can be directed to the corresponding authors.

## Ethics Statement

The animal study was reviewed and approved by The Animal Care Committee of Shandong University (No. ECAESDUSM 20123011).

## Author Contributions

WL and LX designed and supervised the project. XW, YH, FC, MW, and YX performed the experiments and acquired the data. WL, LX, XW, FC, YX, and HW analyzed the results and performed the statistical analysis. WL, LX, and HW wrote the manuscript. All authors contributed to the article and approved the submitted version.

## Conflict of Interest

The authors declare that the research was conducted in the absence of any commercial or financial relationships that could be construed as a potential conflict of interest.

## Publisher’s Note

All claims expressed in this article are solely those of the authors and do not necessarily represent those of their affiliated organizations, or those of the publisher, the editors and the reviewers. Any product that may be evaluated in this article, or claim that may be made by its manufacturer, is not guaranteed or endorsed by the publisher.

## Supplementary Material

Supplementary Figure 1 GPX1 expression was absent in the cochlear SGNs of *gpx1*^–/–^ mice. Immunofluorescence staining and western blot results verified that GPX1 expression was absent in the cochlear SGNs of *gpx1*^–/–^ mice. Cell counting and statistical analysis showed that there was no significant difference in SGNs number between the *gpx1*^–/–^ mice and WT mice. ***p* < 0.01. Scale bar = 12.5 μm.

Supplementary Figure 2 Dose responses of BAY11-7082 co-treated with peroxynitrite in cultured SGNs. The cultured cochlear SGNs were treated with different doses of BAY11-7082 (5 μM, 10 μM and 20 μM) and co-treated with 100 μM peroxynitrite for 48 h. **(A,B)** Immunostaining and cell counting results showed that co-treatment with 10 μM BAY11-7082 effectively increased the number of surviving SGNs after peroxynitrite damage, while the co-treatment with 5 μM or 20 μM BAY11-7082 showed no significant differences in SGN number compared to the peroxynitrite-only group. All data are presented as the mean ± SEM, ** p* < 0.05, *** p* < 0.01. Scale bars: 25 μm.

Supplementary Figure 3 Effects of ebselen on expressions of GPX2 and GPX4 in SGNs after peroxynitrite injury. **(A,C)** Immunofluorescence staining was performed on cochlear frozen cryosection to determine the expression of GPX2 and GPX4 in P3 C57BL/6 mouse cochlea. The Immunofluorescence labeling of GPX2 **(A)** and GPX4 **(C)** was observed in cochlear SGNs of P3 mice. Scale bars: 25 μm. **(B,D)** The cultured middle turn cochleae were treated with peroxynitrite (100 μM) alone, or cotreated with ebselen (30 μM) for 48 h. The immunostaining result showed that the fluorescence intensity of GPX2 **(B)** and GPX4 **(D)** in the peroxynitrite group was reduced compared to that of the control group, while it was increased in the peroxynitrite + ebselen group compared with the peroxynitrite-only group. Scale bars: 12.5 μm.
