# Supplementary figures and images for: Glutathione Peroxidase 1 Protects Against Peroxynitrite-Induced Spiral Ganglion Neuron Damage Through Attenuating NF-κB Pathway Activation
Source: Front Cell Neurosci. 2022 Mar 23;16:841731. doi: 10.3389/fncel.2022.841731 (PMC8983938; doi:10.3389/fncel.2022.841731)

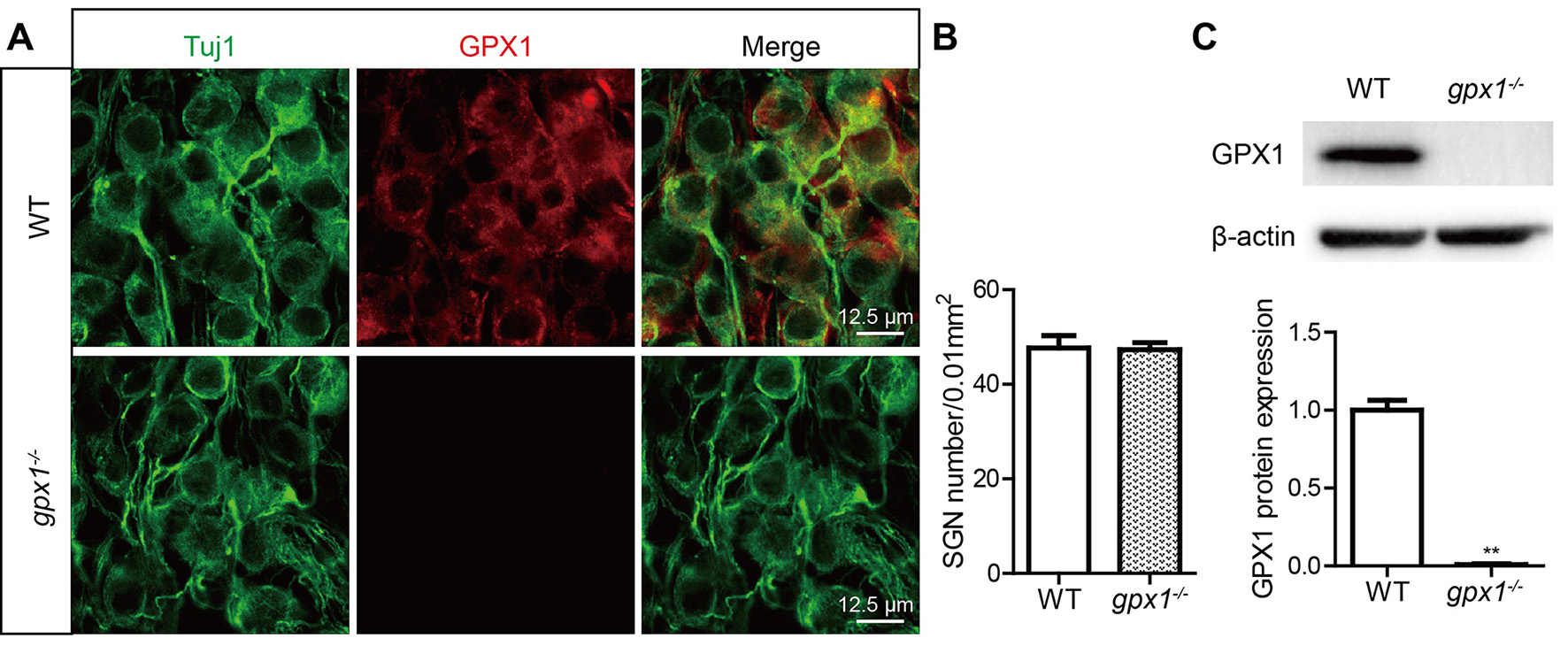

Supplement: Supplementary Figure 1 — GPX1 expression was absent in the cochlear SGNs of gpx1–/– mice. Immunofluorescence staining and western blot results verified that GPX1 expression was absent in the cochlear SGNs of gpx1–/– mice. Cell counting and statistical analysis showed that there was no significant difference in SGNs number between the gpx1–/– mice and WT mice. **p < 0.01. Scale bar = 12.5 μm. [file Image_1.JPEG]

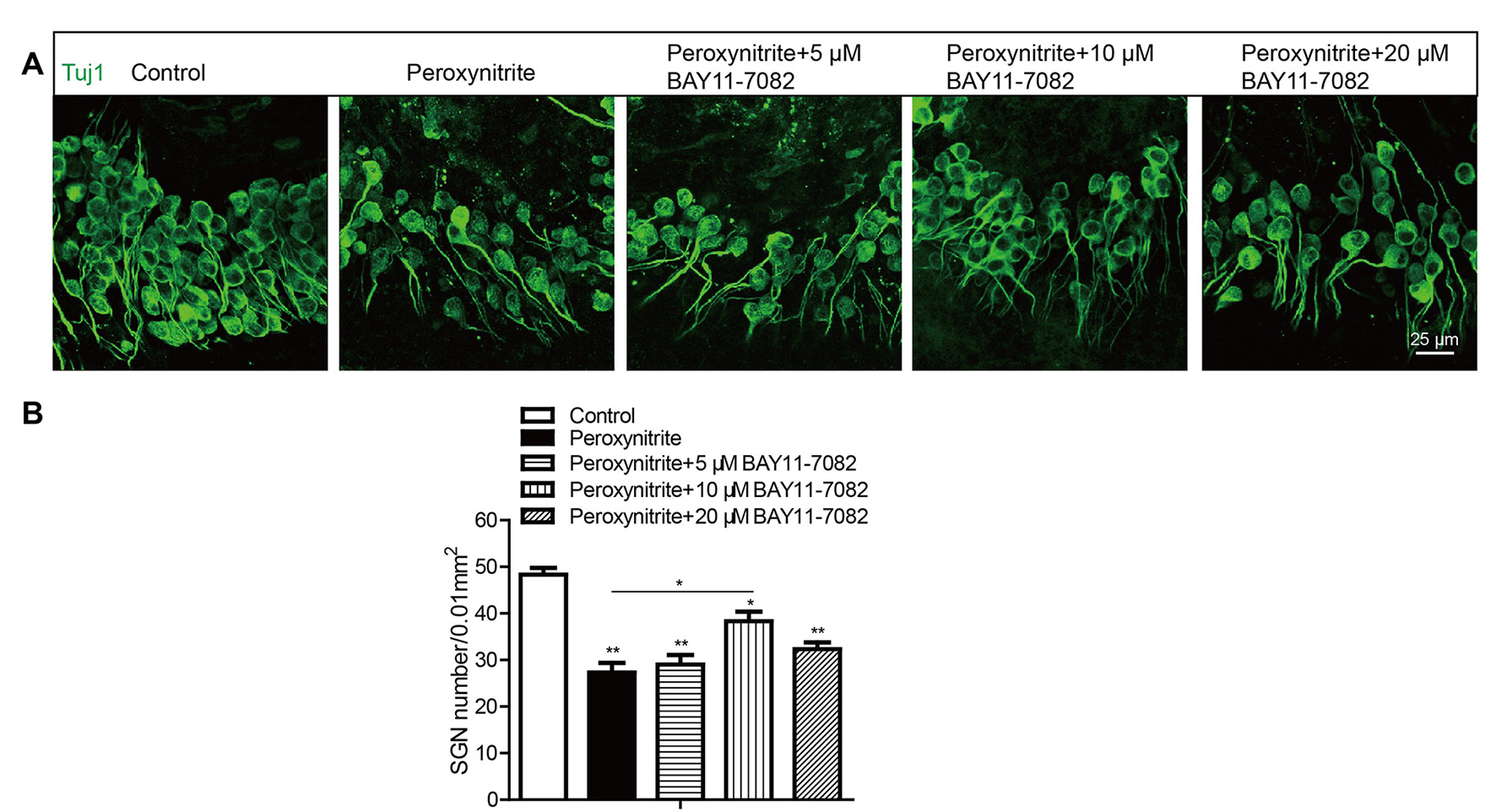

Supplement: Supplementary Figure 2 — Dose responses of BAY11-7082 co-treated with peroxynitrite in cultured SGNs. The cultured cochlear SGNs were treated with different doses of BAY11-7082 (5 μM, 10 μM and 20 μM) and co-treated with 100 μM peroxynitrite for 48 h. (A,B) Immunostaining and cell counting results showed that co-treatment with 10 μM BAY11-7082 effectively increased the number of surviving SGNs after peroxynitrite damage, while the co-treatment with 5 μM or 20 μM BAY11-7082 showed no significant differences in SGN number compared to the peroxynitrite-only group. All data are presented as the mean ± SEM, * p < 0.05, ** p < 0.01. Scale bars: 25 μm. [file Image_2.JPEG]

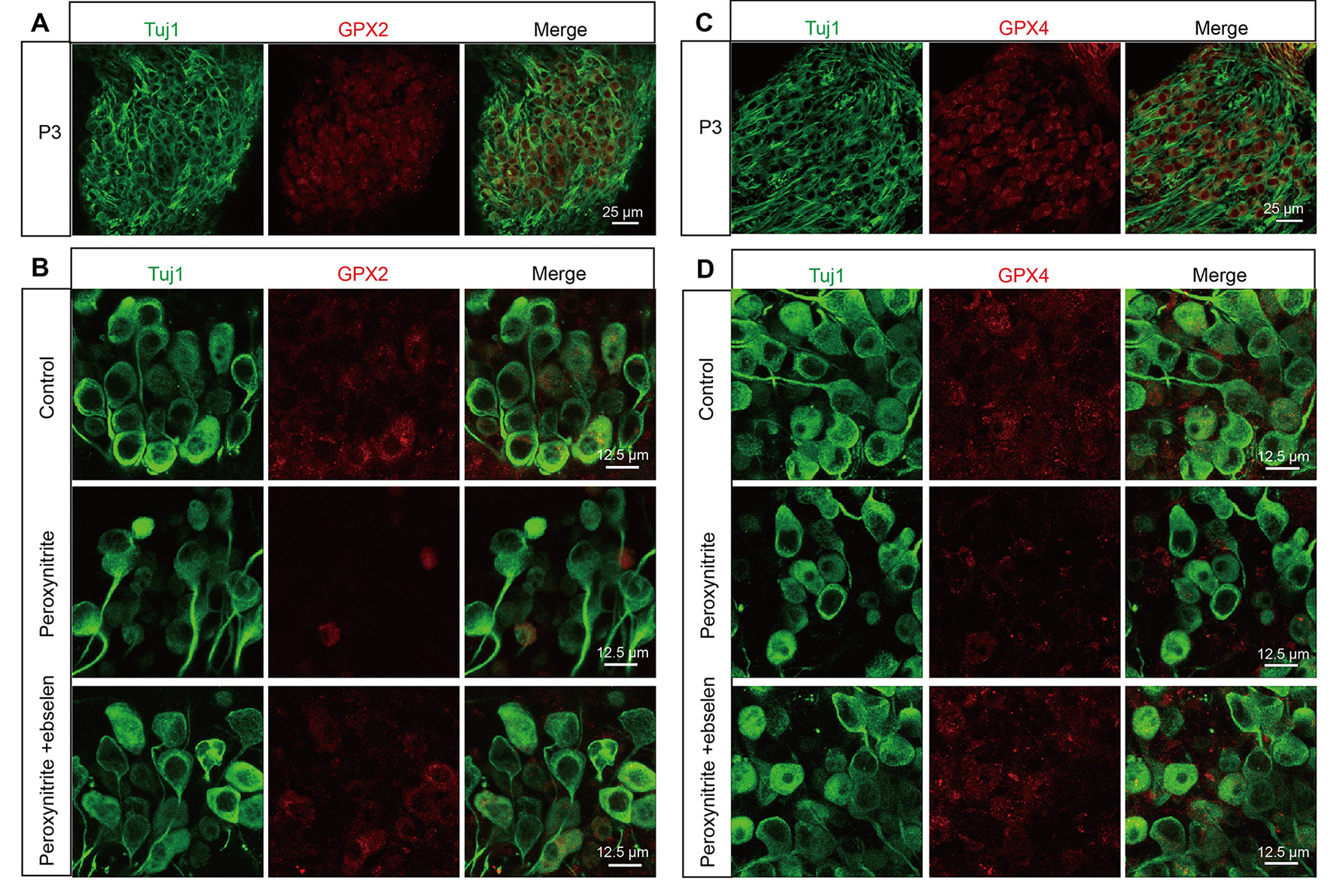

Supplement: Supplementary Figure 3 — Effects of ebselen on expressions of GPX2 and GPX4 in SGNs after peroxynitrite injury. (A,C) Immunofluorescence staining was performed on cochlear frozen cryosection to determine the expression of GPX2 and GPX4 in P3 C57BL/6 mouse cochlea. The Immunofluorescence labeling of GPX2 (A) and GPX4 (C) was observed in cochlear SGNs of P3 mice. Scale bars: 25 μm. (B,D) The cultured middle turn cochleae were treated with peroxynitrite (100 μM) alone, or cotreated with ebselen (30 μM) for 48 h. The immunostaining result showed that the fluorescence intensity of GPX2 (B) and GPX4 (D) in the peroxynitrite group was reduced compared to that of the control group, while it was increased in the peroxynitrite + ebselen group compared with the peroxynitrite-only group. Scale bars: 12.5 μm. [file Image_3.JPEG]
